# 2442. Etiology of Leukemoid Reaction in Patients Undergoing Extracorporeal Membrane Oxygenation

**DOI:** 10.1093/ofid/ofad500.2061

**Published:** 2023-11-27

**Authors:** Christian Wells, Michal Sobieszczyk, Joseph Marcus

**Affiliations:** Brooke Army Medical Center, San Antonio, Texas; Brooke Army Medical Center, San Antonio, Texas; Brooke Army Medical Center, San Antonio, Texas

## Abstract

**Background:**

Infections are a common in patients undergoing extracorporeal membrane oxygenation (ECMO), yet there is limited literature describing best practices for identifying infections. Leukemoid reaction, defined as > 50,000 white blood cells (WBC) per microliter, has been associated with infection in some populations. However, the etiology of leukemoid reactions in ECMO is unknown.

**Methods:**

A retrospective cohort study was performed of all adult patients admitted to Brooke Army Medical Center who received ECMO for greater than 72 hours between the years of 2018-2022. Maximum WBC was obtained for all charts. For patients with a leukemoid reaction, charts were examined to determine both demographic information as well as clinical management of patient at time of leukemoid reaction.

**Results:**

Among 182 patients receiving ECMO for greater than 72 hours, 181 (99%) of patients had a WBC greater than 10,000 cells per microliter during their ECMO course. 15 (8%) developed a leukemoid reaction while on ECMO and were included for further analysis. In this cohort, the median age was 38 [32-44.5] with median [IQR] WBC of 53.94 [50.98-62.55]. Among these patients, 14 (93%) underwent an infectious workup with infectious disease being consulted in 10 (67%) cases. Two (13%) patients had antibiotics started and 2 (13%) had their antibiotics broadened. Patients had a median of 2 [IQR: 2-3] contributing factors to their leukemoid reaction. Of these contributing etiologies, eleven (73%) patients were receiving treatment for a known infection, 6 (40%) were found to have a new thrombus, and 4 (27%) were receiving glucocorticoids (**Table**). One (7%) patient was found to have a new infection, an *Acinetobacter* bacteremia.

**Table 3.**
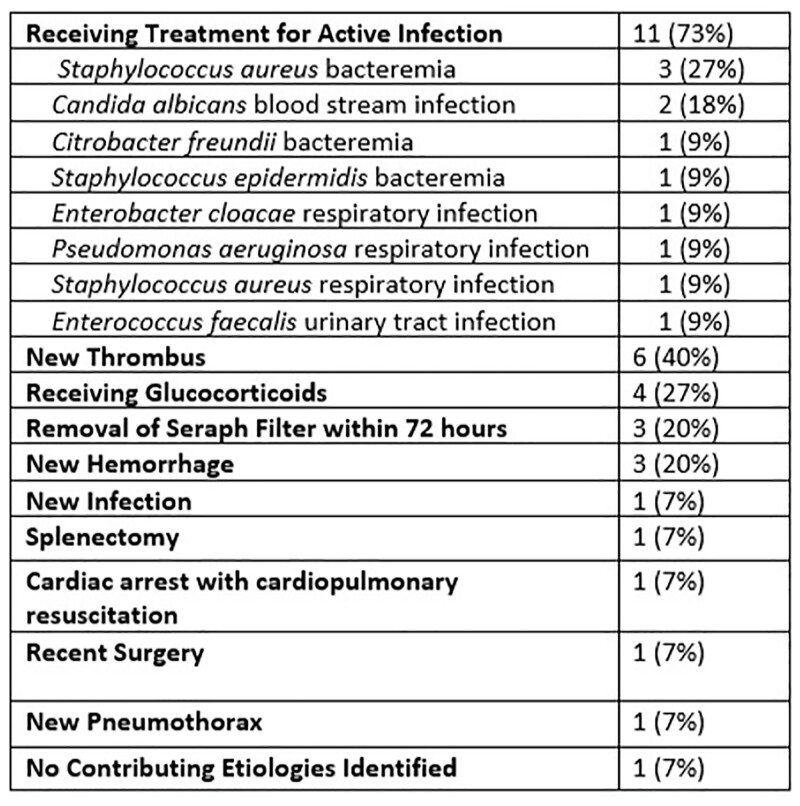
Contributing Etiologies of Leukemoid Reaction in patients receiving ECMO at Brooke Army Medical Center

**Conclusion:**

Leukocytosis is almost universal in ECMO and leukemoid reactions occur frequently. In this cohort, infectious workups were common for leukemoid reaction, yet new infections were rarely identified. Future studies are needed to determine better infectious markers in this patient population.

**Disclosures:**

**All Authors**: No reported disclosures

